# β-Cyclodextrin Polymer-Based Host–Guest Interaction and Fluorescence Enhancement of Pyrene for Sensitive Isocarbophos Detection

**DOI:** 10.1021/acsomega.1c07295

**Published:** 2022-04-08

**Authors:** Shanshan Gao, Gege Yang, Xiaohui Zhang, Ying Lu, Ying Chen, Xiangwei Wu, Chunxia Song

**Affiliations:** † Department of Applied Chemistry, School of Science, Anhui Agricultural University, Hefei 230036, China; ‡ College of Resources and Environment, Key Laboratory of Agri-food Safety of Anhui Province, Anhui Agricultural University, Hefei 230036, China

## Abstract

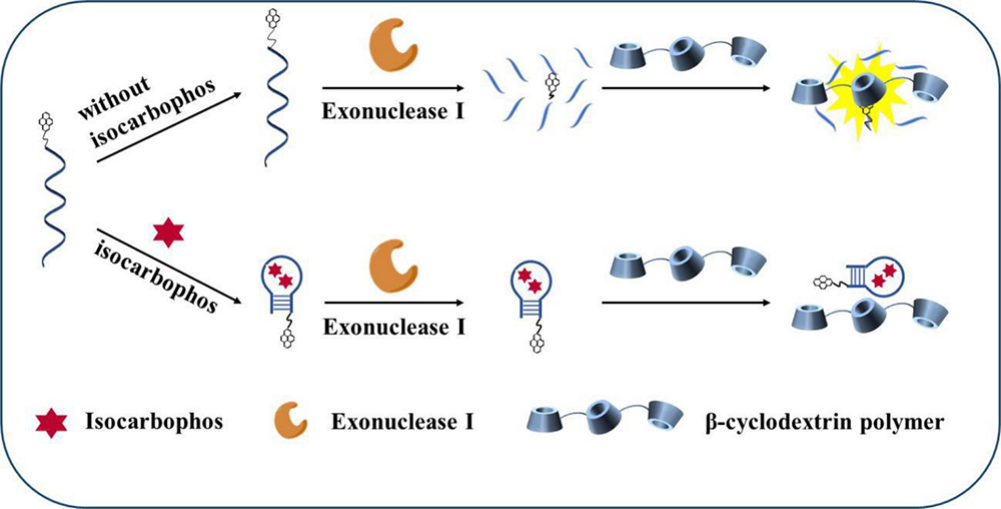

The extensive use of organophosphorus pesticides in agriculture poses a high risk to human health and has boosted the demands for developing sensitive monitoring methods. Herein, we developed a facile and sensitive method for isocarbophos detection based on the remarkable fluorescence enhancement of pyrene during host–guest interaction of β-cyclodextrin polymer (β-CDP) and pyrene. The 3′-pyrene-labeled isocarbophos aptamer could be cleaved by exonuclease I to obtain free pyrene that was tagged on mononucleotides, which could enter the hydrophobic cavity of β-CDP, resulting in a prominent fluorescence enhancement. While the target isocarbophos was added, aptamer could undergo a conformational change into a hairpin complex, which prevented the cleavage and host–guest interaction because of the steric hindrance, leading to a weak fluorescence. The isocarbophos has been sensitively and selectively analyzed by detecting the system fluorescence intensity with a detection limit as low as 1.2 μg/L. In addition, we have verified the ability of our proposed method in real sample detection from fruit extract.

## Introduction

Organophosphorus pesticides have been widely applied throughout the world for their high efficiency in preventing diseases and pests from harming crops.^1−3^ Nevertheless, they are highly neurotoxic because of their inhibition effect on acetylcholinesterase activity in the central and peripheral nervous system. More severely, the continual assimilation by plants and enrichment in the food chain mean that even a very low concentration of organophosphorus pesticides could threaten human health, which raises people’s concern about their health effects.^4,5^ There have been a large number of reports about organophosphorus pesticides being related to human diseases such as attention deficit hyperactivity disorder, Parkinson’s, amyotrophic lateral sclerosis, Alzheimer’s, chronic diseases of the central nervous system, etc.^6^ Accordingly, it is urgent to develop highly sensitive methods for organophosphorus pesticide analysis in the field of food safety protection.

Up to now, chromatography, ultraviolet–visible spectroscopy, electrochemical technology, capillary electrophoresis, fluorescence methods, etc.,^7−15^ have been widely used in the detection of organophosphorus pesticides. Among them, fluorescence methods have aroused great interest because of their simple operation, low sample usage, and easy readout and quantification.^16,17^ However, many fluorometric immunoassays that are based on antibodies as the recognition department suffer from the high cost and instability of antibodies, which limits their wide application. At present, aptamers have been widely used as artificial antibodies in fluorescence methods because of their high affinity, excellent stability, and prolonged storage life.^18,19^ For example, Dou et al. have established a gold-based nanobeacon fluorescence probe for organophosphorus pesticide sensing, with a detection limit of quantification reaching 10 μg/L.^16^ In order to monitor trace-level organophosphorus pesticide residuals and overcome the current issue of severe pollution, it is urgent to develop highly sensitive fluorescence methods.

Because of their unique properties compared to the properties of monomers, polymers such as fluorescent conjugated polymers, molecularly imprinted polymers, nucleic acid, etc., have had widespread use as fundamental materials in analysis fields.^20−23^ Cyclodextrin polymer is constituted of monomer cyclodextrin, the most commonly used host molecule, through polymerization, graft copolymerization, or molecular cross-linking. It not only retains the characteristics of highly specific host–guest recognition of monomer cyclodextrin^24,25^ but also exhibits some advantages like good solubility, high stability, a cross-linking agent effect, and a multivalent binding effect. More importantly, Hollas et al. reported that cyclodextrin polymer has shown a stronger fluorescence enhancement effect for fluorophores than monomer cyclodextrin because of the increase in the overall complexation constant by more than 2 orders.^26^ Based on these excellent characteristics of β-cyclodextrin polymer, we have developed highly sensitive detection methods for biological small molecules, enzymes, nucleic acids, etc.^27,28^

**Scheme 1:**
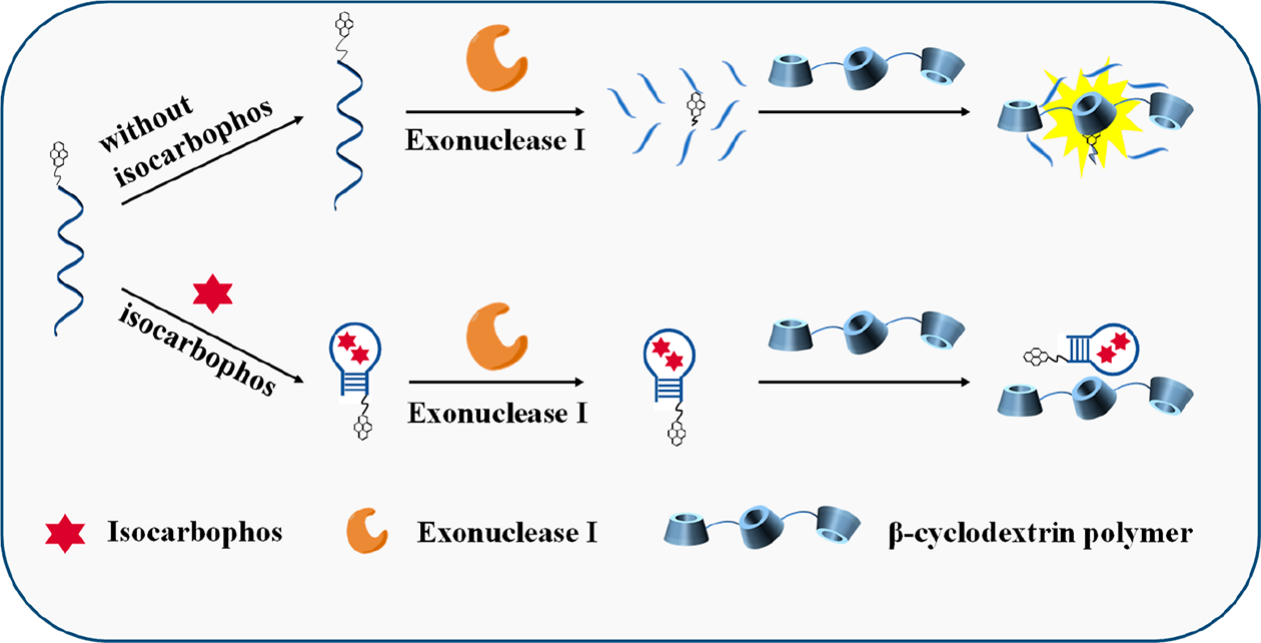
Schematic Representation of β-Cyclodextrin Polymer–Based Host–Guest Interaction and Fluorescence Enhancement of Pyrene for Isocarbophos Monitoring with High Sensitivity

Inspired by these strategies, we intend to design a highly sensitive method for isocarbophos detection based on a prominent fluorescent enhancement of cyclodextrin polymer combined with the highly selective recognition ability of the nucleic acid aptamer. As shown in Scheme 1, pyrene was single-labeled on the 3′-terminal of the isocarbophos DNA aptamer, which was inexpensively purchased from TaKaRa Bio. Inc. (Dalian, China) with high stability. The exonuclease I (Exo I) could digest aptamer to produce pyrene tagged on mononucleotides, and it was easy for the pyrene tagged on mononucleotides to enter the cavity of β-CDP, accompanied by a prominent fluorescence enhancement. In the presence of target isocarbophos, the aptamer could combine with isocarbophos and then fold into a hairpin complex, with four pairs of bases positioned on its ends, so Exo I could not digest it. The steric hindrance of the hairpin complex impeded the interaction of pyrene and β-CDP, which affected the fluorescence enhancement. The fluorescence of pyrene decreased with the increase in isocarbophos concentration. Therefore, a fluorescence method with high sensitivity and convenience for quantitative isocarbophos monitoring could be achieved through the relationship between the fluorescence signal and isocarbophos.

## Results and Discussion

### Prominent Fluorescence Enhancement of β-CDP

β-CDP was synthesized according to the literature.^29^ The results of Fourier transform infrared spectroscopy (Figure S1), the particle size distribution (Figure S2), and the ζ potential diagram (Figure S3) proved that β-CDP had been successfully synthesized. The detailed experimental procedures and characterization are described in the Supporting Information.

**Figure 1:**
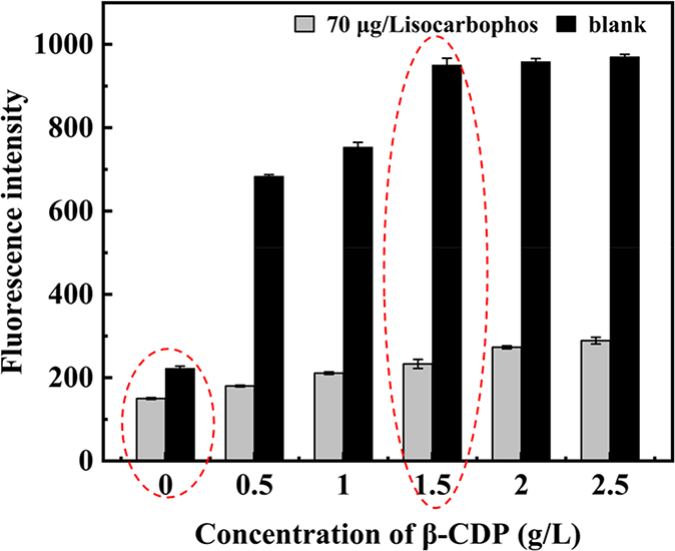
Fluorescence responses while the concentration of β-CDP was changed from 0 to 2.5 g/L, the concentration of isocarbophos was 70 μg/L. The concentration of aptamer and activity of Exo I were 400 nM and 140 U/mL, respectively. The emission wavelength was set at 345 nm. Error bars indicate the standard deviations of three experiments.

To verify the efficient fluorescence enhancement capability of β-CDP, we investigated the fluorescence response with the concentration of β-CDP changing from 0 to 2.5 g/L. In the presence of β-CDP and isocarbophos, the fluorescence of the system remained weak because DNA aptamer labeled with pyrene could specifically recognize the isocarbophos to form a hairpin structure, which prevented DNA aptamer from being digested by Exo I, and it hardly entered the cavity of β-CDP (Figure 1). While in the absence of isocarbophos, the free pyrene attached on mononucleotides could be obtained through the digestion of aptamer by Exo I. The addition of β-CDP from 0.5 to 2.5 g/L resulted in a great fluorescence intensity enhancement. The fluorescence could be increased by more than 4 times in the presence of 1.5 g/L β-CDP, which was used for further study.

### Isocarbophos-Induced Protection of 3′-Pyrene-Labeled Aptamer

**Figure 2:**
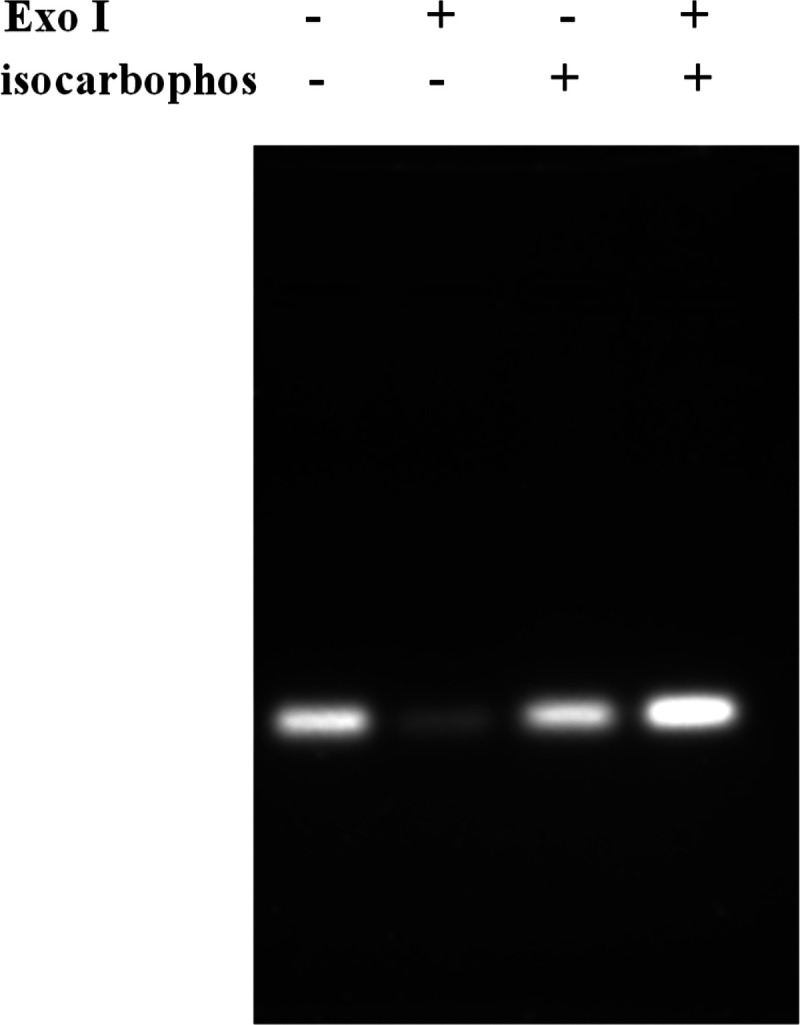
Agarose gel electrophoresis.

Gel electrophoresis was further used to demonstrate that isocarbophos could protect 3′-pyrene-labeled aptamer from detachment. As shown in Figure 2, as for the first and third lanes (from the left to right), bright bands were obtained in the absence of Exo I, which proved that no aptamer detached, while in the presence of Exo I (the second and fourth lanes), there was no distinct band observed in the case without isocarbophos (the second lane). Conversely, a bright band was obtained in the presence of isocarbophos (the fourth lane), indicating that the aptamer was protected from digestion by isocarbophos.

### Analytical Performance

**Figure 3:**
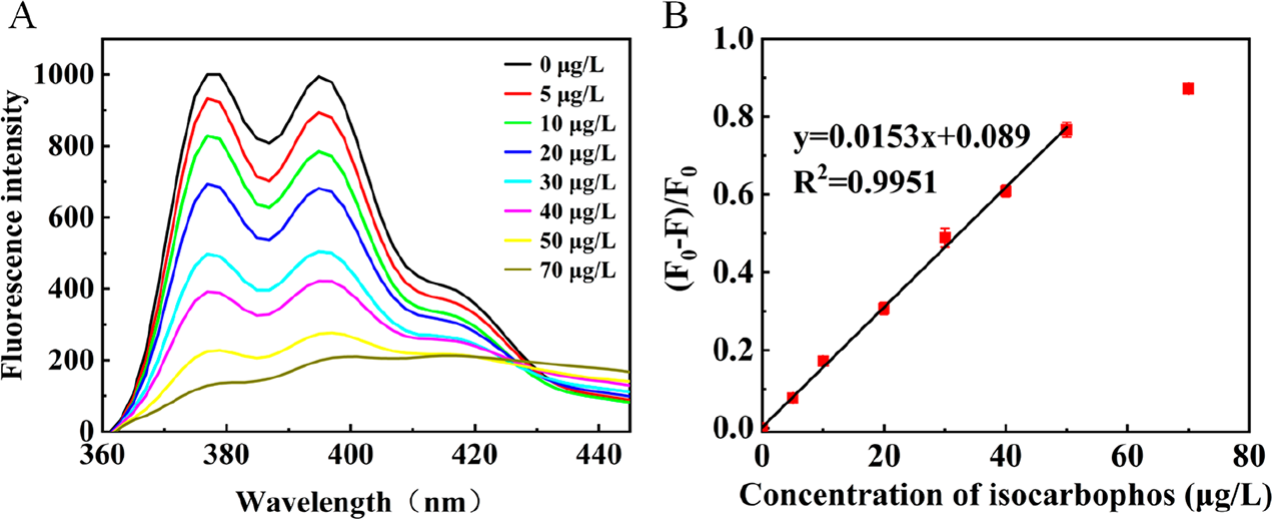
(A) Fluorescence spectra of the system with different concentrations of isocarbophos. The concentration of isocarbophos from bottom to top: 0, 5, 10, 20, 30, 40, 50, and 70 μg/L. (B) Linear relation plots of (*F*_0_ – *F*)/*F*_0_ vs isocarbophos concentration in the range 0–50 μg/L. The concentrations of Exo I, aptamer, and β-CDP were 140 U/mL, 400 nM, and 1.5 g/L, respectively. The emission wavelength was set at 345 nm. Error bars indicate the standard deviations for three experiments.

In order to verify the ability of the strategy for quantitative detection, a concentration gradient experiment of isocarbophos was carried out under optimal conditions (Figure S4 in the Supporting Information details the optimization of experimental conditions). Figure 3A demonstrates that the fluorescence emission was decreased with the increase of isocarbophos. As depicted in Figure 3B, (*F*_0_ – *F*)/*F*_0_ (*F*_0_ and *F* represent the fluorescence responses of this method without and with isocarbophos) increased linearly with the concentration of isocarbophos in the range 0–50 μg/L (*y* = 0.0153*x* + 0.089, *R*^2^ = 0.9951). In addition, this method showed a low detection limit (LOD = 1.2 μg/L, S/N = 3). Table 1 demonstrates that the detection limit of our method was competitive compared to some reported approaches. The high sensitivity of this method was attributed to the prominent fluorescence enhancement capability of β-CDP. Moreover, the β-CDP was weakly electronegative (the ζ potential diagram was −15 Mv, Figure S3). The DNA aptamer was also negatively charged. The mutually repulsive force between β-CDP and the aptamer could ensure the low background of this method and therefore contributed to the high sensitivity.

**Table 1:** Performance Comparison between Our Constructed Method and Reported Isocarbophos Detection Methods

methods	tools	detection limit (μg/L)	ref
magnetic solid phase extraction	magnetic graphene nanocomposite	33	(30)
fluorescence	gold-based nanobeacon probe	10	(16)
capillary electrophoresis	quantum dot–DNA aptamer conjugates	49.13	(14)
Raman scattering method	single aptamer	144.5	(31)
fluorescence	fluorescence polarization aptamer assay	5.0	(32)
fluorescence	β-cyclodextrin polymer-based fluorescence enhancement	1.2	This work

### Selectivity of Our Proposed Approach

**Figure 4:**
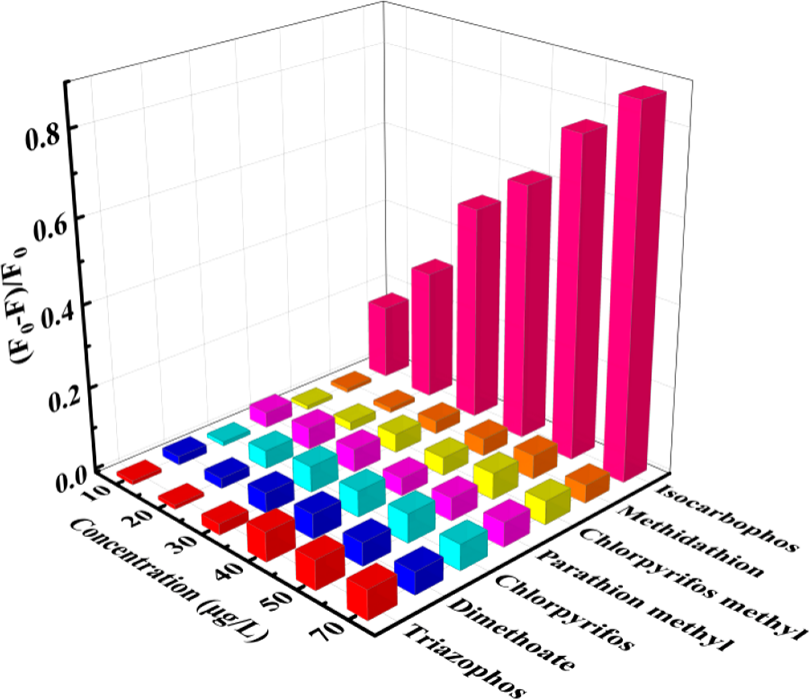
Fluorescence signals of the detection system in the cases of different concentrations of triazophos, dimethoate, chlorpyrifos, parathion methyl, chlorpyrifos methyl, methidathion, or isocarbophos. The *X*-axis, *Y*-axis, and *Z*-axis represent concentration gradients, different interferences, and (*F*_0_ – *F*)/*F*_0_, respectively.

For a further evaluation of the selectivity of this method, 6 potential interferents of other organophosphorus pesticides were investigated. As depicted in Figure 4, the concentration of the maximum signal (*F*_0_ – *F*)/*F*_0_ was 40 μg/L for triazophos, 50 μg/L for dimethoate, 50 μg/L for chlorpyrifos, 70 μg/L for parathion methyl, 50 μg/L for chlorpyrifos methyl, and 50 μg/L for methidathion. For these potential interferents, the maximum signals of (*F*_0_ – *F*)/*F*_0_ were below 0.10. However, only the addition of isocarbophos could trigger a large signal of (*F*_0_ – *F*)/*F*_0_; the maximum signal of (*F*_0_ – *F*)/*F*_0_ in the case of isocarbophos was close to 0.9. These results verified that our constructed method has excellent selectivity.

### Real Sample Detection

Our proposed method was used to analyze residual isocarbophos in apples to demonstrate the strategy in the application of real samples. The apple extract was prepared according to a report^33^ with minor alteration. Then, different standard concentrations of isocarbophos were added into the apple extract. Table 2 demonstrated that the average recovery values from 92.2% to 103.6% were obtained with a relative standard deviation (RSD) within 10.9 (*n* = 3) from the actual apple samples spiked with our proposed method. While using GC analysis to detect isocarbophos, the recoveries were in the range 79.3–105.3%, with a much higher detection limit of 28.9 μg/L than our first spiked sample, 5 μg/L.^15^ These results proved the accuracy and reliability of our established method in food safety applications.

**Table 2:** Recovery Results of Real Apple Samples Analyzed through Our Proposed Method

sample	spiked isocarbophos (μg/L)	average detected isocarbophos (*n* = 3, μg/L)	recovery (%)	RSD (*n* = 3) (%)
1	5	4.69	93.8	10.9
2	10	10.27	102.7	7.9
3	20	18.44	92.2	2.4
4	40	40.88	102.2	5.6
5	50	51.80	103.6	8.4

## Conclusion

In conclusion, a highly sensitive and facile fluorescence method for isocarbophos monitoring has been constructed that combines the significant fluorescence enhancement of β-CDP with the high-specificity/affinity aptamer. Highly sensitive detection of isocarbophos was achieved due to the remarkable fluorescence enhancement ability of β-CDP with the wide range 5–50 μg/L and a detection limit as low as 1.2 μg/L. Additionally, the high selectivity was attributed to the ultraselective aptamer with high affinity. Moreover, it was successfully used to determine residual isocarbophos in fruit samples. Altogether, the construction of this method offers a new choice for highly sensitive and selective organophosphorus pesticide detection and has great application potential in the field of food safety inspection.

## Materials and Methods

### Materials

Isocarbophos (99%, purity) was brought from Shanghai Pesticide Research Institute (Shanghai, China), and chlorpyrifos (98%) was provided by Sinopharm Chemical Reagent Co., Ltd. (Shanghai, China). Acetone was purchased from Xilong Scientific Co., Ltd. (Shantou, China). A 3′-pyrene-labeled isocarbophos DNA aptamer (5′-AGCTTGCTGCAGCGATTCTTGATCGCCACAGAG CT-3′) was synthesized by TaKaRa Bio. Inc. (Dalian, China). β-Cyclodextrin was bought from Sigma-Aldrich Co., Ltd. Tris hydroxymethyl aminomethane, toluene, epichlorohydrin, isopropanol, and sodium hydroxide were obtained from China National Pharmaceutical Group Co., Ltd. 10× Exo I buffer (67 mM MgCl_2_, 670 mM glycine–KOH, 10 mM dithiothreitol, pH = 9.5) and Exo I were provided by Sangon Biotech Co., Ltd. (Shanghai, China). The ultrapure water was provided by a Millipore water purification system (≥18.2 MΩ cm).

### Isocarbophos Detection

Exo I buffer solution (10×, 30 μL) was added into 208 μL of sterilized ultrapure water and 30 μL of 4 μM DNA aptamer solution in a centrifuge tube. Then, 42 μL of isocarbophos standard solution was added to the mixed solution. Subsequently, 140 units of Exo I was mixed into the system and incubated at 37 °C for 30 min in a dry bath, followed by heating for 20 min at 80 °C to inactivate Exo I, and the mixture was then allowed to cool to 25 °C. Afterward, 30 μL of 15 g/L β-CDP was added into the centrifuge tube. Lastly, the fluorescence signals of the system were detected with an Agilent G9800A fluorescence spectrophotometer. The excitation wavelength was 345 nm, and the scanning range of the fluorescence emission spectrum was 350–500 nm, with the excitation set at 5 nm and emission slit at 10 nm.

### Agarose Gel Electrophoresis Experiment

Agarose powder (0.5 g) was mixed into 25 mL of 1× TAE electrophoresis buffer (1 mM Na_2_EDTA, 40 mM Tris-acetate, pH 8) to prepare an agarose gel (2%). A 2 μL portion of Sybr Gold and 2 μL of loading buffer were mixed with 10 μL of the sample, and the mixture was then added to the agarose gel for electrophoresis experiments. In the same TAE buffer, electrophoresis was performed at 100 V for 30 min, and then, the gel was photographed using gel imaging under ultraviolet light.

### Pretreatment of Apple Samples

We chose fresh apples as the practical samples to inspect the application potential of our constructed method, which were bought from a supermarket (Hefei, China). At first, 20 mL of freshly squeezed apple juice was mixed with 40 mL of acetone and 10 mL of water, and the mixture was allowed to sit for 10 min. Then, the mixture was filtrated with a 0.22 μm filter membrane after remaining in an ultrasonic bath for 30 min. Afterward, we concentrated the filtrate in rotary evaporators at 40 °C. Finally, the solution product was redissolved in 10× Exo I buffer with a ratio of 1:100.

## Supporting Information Available

Preparation of β-cyclodextrin polymer; characterization of β-cyclodextrin polymer; and optimization of experimental conditions (PDF)

## Abbreviations

β-CDP: β-cyclodextrin polymer
Exo I: exonuclease I
RSD: relative standard deviation
