# Supplementary material for: β-Cyclodextrin Polymer-Based Host–Guest Interaction and Fluorescence Enhancement of Pyrene for Sensitive Isocarbophos Detection
Source: ACS Omega. 2022 Apr 8;7(15):12747–52. doi: 10.1021/acsomega.1c07295 (PMC9026021; doi:10.1021/acsomega.1c07295)
Supplement: Supplementary file 1 — ao1c07295_si_001.pdf [file ao1c07295_si_001.pdf]

Electronic Supplementary Information

β-cyclodextrin polymer based host-guest interaction and fluorescence enhancement of pyrene for sensitive isocarbophos detection

Shanshan Gao[1], Gege Yang[1], Xiaohui Zhang[1], Ying Lu[1], Ying Chen[1], Xiangwei Wu[*2], and Chunxia Song[*1]

[1]*Department of Applied Chemistry, School of Science, Anhui Agricultural University*

[2]*College of Resources and Environment, Key Laboratory of Agri-food Safety of Anhui Province, Anhui Agricultural University*

[*]Corresponding Authors: E-mails: songchunxia@ahau.edu.cn (C. S.), wxw@ahau.edu.cn (X. W.)

Experimental details

1. Preparation of β-cyclodextrin polymer.

2. Characterization of β-cyclodextrin polymer.

3. Optimization of experimental conditions.

1. **Preparation of β-cyclodextrin Polymer (β-CDP)**

The β-CDP was synthesized according to our previous work with minor modification[1]. Firstly, 10 g (8.81 mmol) β-CD was dissolved in 30 mL 15% NaOH aqueous solution, and stirred for 2 h in 35 °C. Secondly, 2 mL (8.81 mmol) methylbenzene was added to 2 h. Thirdly, 8.81 mmol epichlorhydrin was added drop by drop and stirred 3 h of at 35 °C. 200 mL isopropanol was added to the mixture and 6 mol/L HCl was used to adjust the pH value of the flocculating constituent to 7.0. Then the mixture was poured into dialysis tube (MWCO 5000~8000) and dialyzed in pure water for 7 days. Finally, the dialysate in the dialysis tube was dried at -60 °C under vacuum overnight. Thereby the white water soluble β-CDP was synthesized.

2. Characterization of β-cyclodextrin Polymer

We further investigated the characterization of β-CDP through Fourier Transform Infrared Spectroscopy (FTIR), particle size distribution and Zeta potential. FTIR spectra of β-CD and β-CDP were shown in **Figure S1**. The epoxy absorption peak at 893 cm^{-1} of β-CDP disappeared, indicating that epichlorohydrin has reacted with β-CD. The peak at 3443 cm^{-1} was obtained for -OH stretching. The absorption peak at 2923 cm^{-1} was assigned to the stretching vibrations of $-CH_2-$. The absorption peak at 1300~1000 cm^{-1} was the characteristic peak of the ether bond. These results indicated that cyclodextrin monomer existed in the cyclodextrin polymer. The average particle size distribution of β-CDP was closed to 207 μm due to the high solution concentration and crosslinking (**Figure S2**). As shown in **Figure S3**, the Zeta potential diagram of β-CDP was -15 mV, indicating that the polymer was weakly electronegative. These results indicated that we have successfully synthesized β-cyclodextrin polymer with weakly negative charged.

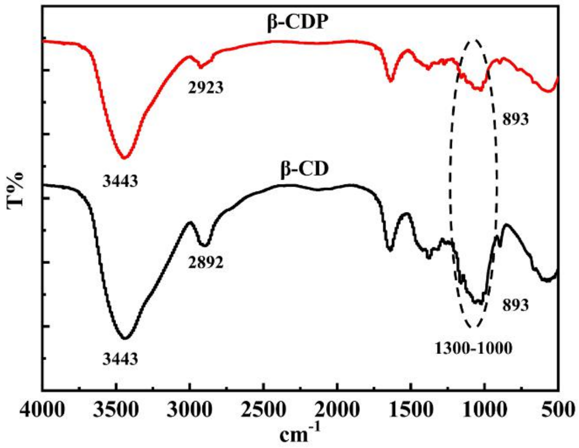

Figure S1. FTIR spectra of β-CD and β-CDP

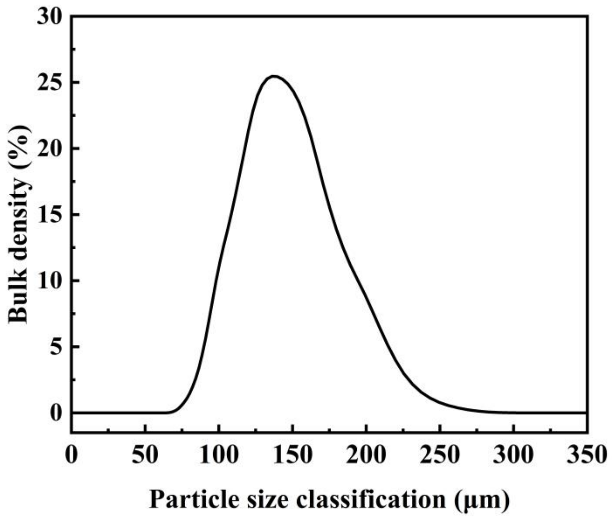

Figure S2. Particle size distribution of β-CDP

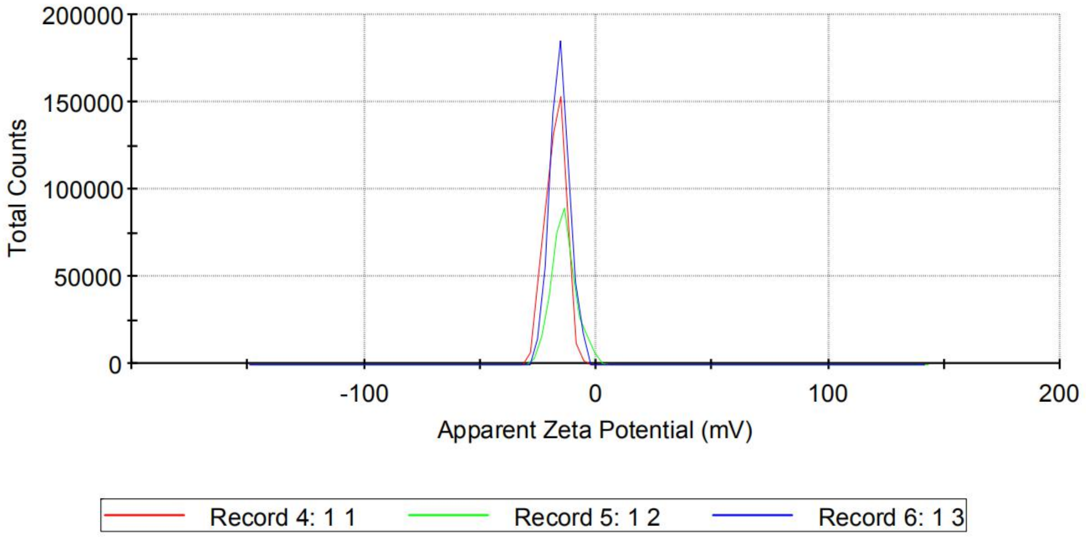

Figure S3. Zeta potential diagram of β-CDP

3. Optimization of Experiment Conditions

In order to obtain ideal analytical performance, the concentrations of aptamer and Exo I were optimized. In this study, $(F_0-F)/F_0$ ratio was used as a standard to select the optimum condition, F_0 and F represents the fluorescence responses intensity of isocarbophos at 0 and 30 μg/L, respectively. As shown in **Figure S4 A**, with the increasing of the aptamer concentration, $(F_0-F)/F_0$ gradually increased and then reached the maximum when 400 nM was used. As shown in **Figure S4 B**, $(F_0-F)/F_0$ achieved the maximum when the concentration of Exo I was 140 U/mL . Therefore, 400 nM aptamer and 140 U/mL Exo I were chosen for further research.

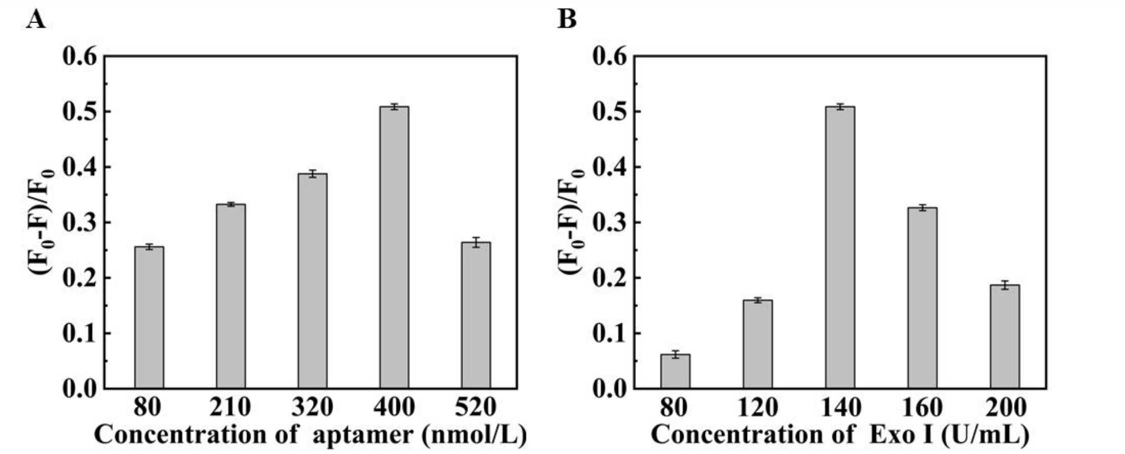

Figure S4. Optimization of experimental conditions. (A) Different concentration of aptamer, the activity of Exo I was 140 U/mL. The concentration of isocarbophos was 30 μg/L. (B) Different activity of Exo I, the concentration of aptamer and isocarbophos was 400 nM and 30 μg/L, respectively. The concentration of β-CDP was 1.5 g/L. The emission wavelength was set at 345 nm. Error bars indicated the standard deviations of three experiments.

REFERENCES

(1) Koopmans, C.; Ritter, H., Formation of Physical Hydrogels Via Host-Guest Interactions of β-Cyclodextrin Polymers and Copolymers Bearing Adamantly Groups. *Macromolecules* **2008**, *41*, 7418-7422.
